# More than a rash: lamotrigine-induced hypersensitivity mimicking DRESS in a bipolar patient with early-life trauma

**DOI:** 10.1192/j.eurpsy.2026.11205

**Published:** 2026-07-31

**Authors:** L. Jishkariani, G. Garuchava, G. Cheishvili

**Affiliations:** 1 Psychiatry, Tbilisi state medical university; 2 medicine, Tbilisi state university, Tbilisi, Georgia

## Abstract

**Introduction:**

Lamotrigine is effective in bipolar depression but carries a risk of severe cutaneous adverse reactions (SCARs), including Drug Reaction with Eosinophilia and Systemic Symptoms (DRESS). Early identification is crucial, especially in vulnerable psychiatric populations. Here, we present a diagnostically complex case of incomplete DRESS in a trauma-exposed adolescent, highlighting the interplay between emotional stress, immune dysregulation, and adverse drug reactions.

**Objectives:**

To (1) describe a rare case of probable lamotrigine-induced DRESS with incomplete criteria; (2) explore how early-life trauma may influence drug reactivity through neuroimmune pathways; and (3) emphasize the role of trauma-informed care in enhancing diagnostic accuracy and treatment safety.

**Methods:**

A 19-year-old female with a history of emotional neglect, early parental separation, and caregiver burden presented with subdepressive symptoms and mixed features. Pharmacological treatment included fluoxetine, quetiapine (PRN), and lamotrigine (titrated from 25 mg QHS). Within two weeks, she developed malaise, low-grade fever, a widespread maculopapular rash, lymphadenopathy, arthralgia, and elevated hepatic transaminases. An extensive workup ruled out infectious and autoimmune etiologies. Clinical photographs were taken to document rash distribution and morphology. RegiSCAR and Japanese criteria were applied to assess DRESS probability.

**Results:**

The patient developed a confluent erythematous maculopapular rash affecting the limbs, trunk, and face, with greater intensity over the knees, forearms, and lower back (see Figures 1–3). Associated symptoms included fatigue, low-grade fever (37.8°C), lymphadenopathy, joint pain, and lab abnormalities (eosinophils 8%, AST 49.6 U/L, ALT 80 U/L). Infectious and autoimmune panels were negative. Although she did not meet full RegiSCAR criteria (fever <38.5°C, eosinophilia <10%), major criteria were fulfilled. Rash and systemic signs resolved after lamotrigine withdrawal and corticosteroid treatment.

**Table 1.** RegiSCAR Criteria vs. Patient Presentation Table 1. Long description.

Criterion	Present?	RegiSCAR Match
Maculopapular rash	Yes	**✓**
Fever >38.5°C	No	❌
Eosinophilia >10%	No	❌
Lymphadenopathy	Yes	**✓**
Liver involvement (ALT↑)	Yes	**✓**
Renal/other organ involvement	Partial	⚠
Duration >15 days	No	❌

**Image 1:**
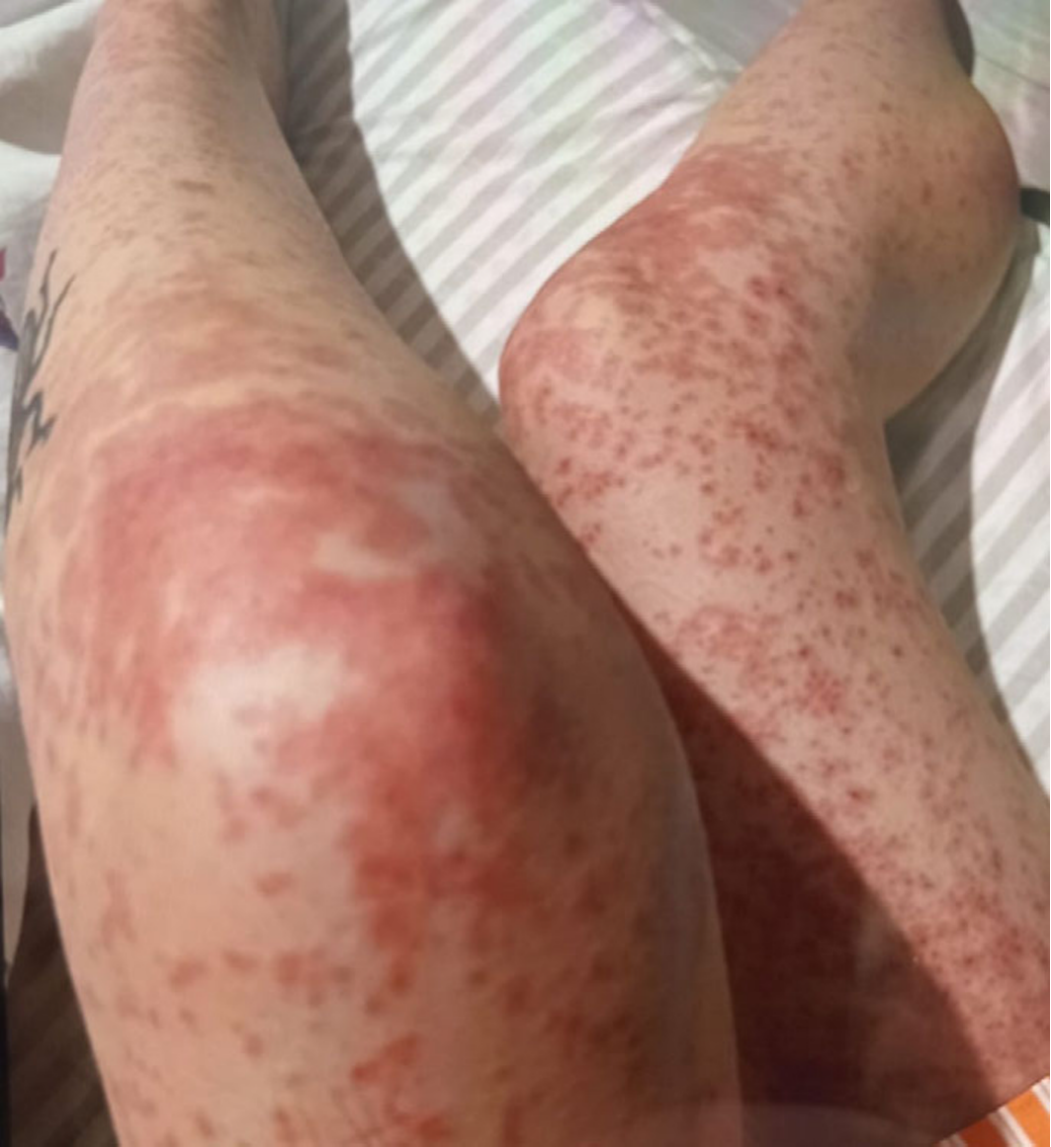

**Image 2:**
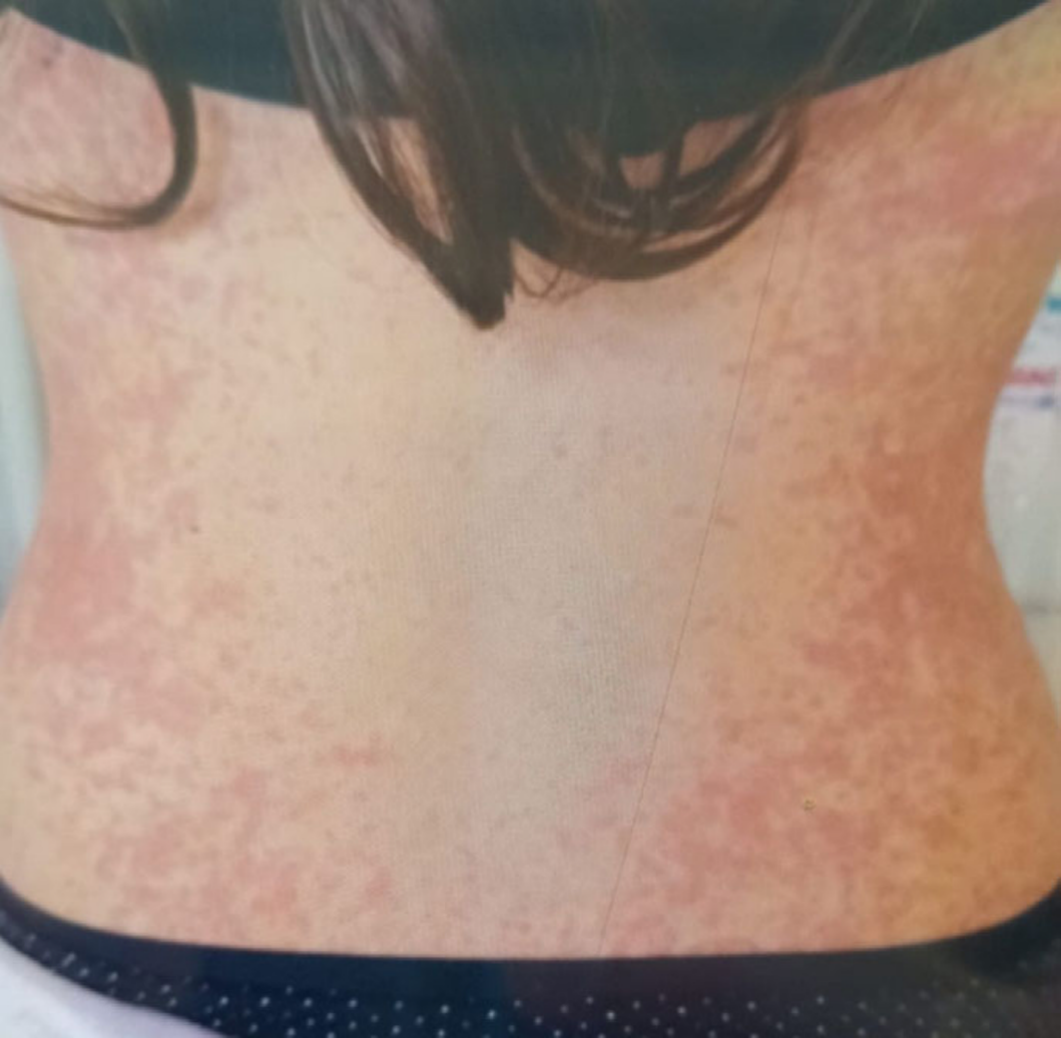

**Image 3:**
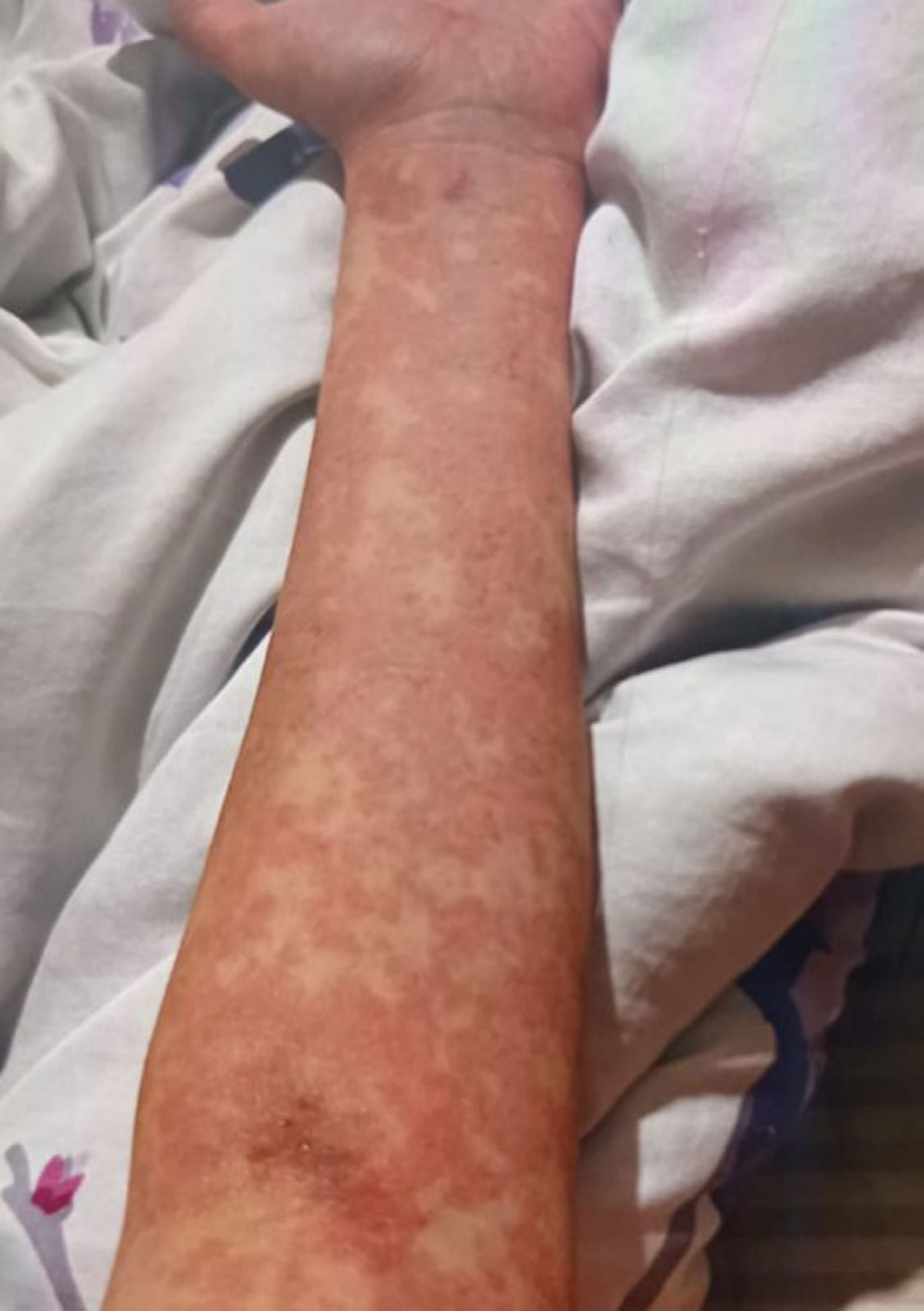

**Conclusions:**

This case illustrates the diagnostic complexity of drug-induced hypersensitivity in the context of early-life trauma. Trauma may act as a sensitizing factor through HPA axis dysregulation and immune priming, lowering the threshold for systemic inflammatory responses. Trauma survivors also exhibit heightened interoceptive awareness, which can amplify symptom perception. Trauma-informed approaches not only improve diagnostic clarity but may also help mitigate the risk of SCARs.

**Disclosure of Interest:**

None Declared

## Long descriptions

### Table 1. Long description

The table consists of seven rows of criteria.

* Row 1: Maculopapular rash. Present? Yes. Regi S C A R Match: Checkmark.

* Row 2: Fever greater than 38.5 degrees Celsius. Present? No. Regi S C A R Match: Red X.

* Row 3: Eosinophilia greater than 10 percent. Present? No. Regi S C A R Match: Red X.

* Row 4: Lymphadenopathy. Present? Yes. Regi S C A R Match: Checkmark.

* Row 5: Liver involvement (A L T up arrow). Present? Yes. Regi S C A R Match: Checkmark.

* Row 6: Renal or other organ involvement. Present? Partial. Regi S C A R Match: Warning symbol.

* Row 7: Duration greater than 15 days. Present? No. Regi S C A R Match: Red X.

Navigate back to Table 1..

